# Physical Recovery After CAR-T Cell Therapy vs. Autologous Stem Cell Transplantation Assessed Through Quantitative Rehabilitation Measures

**DOI:** 10.3390/biomedicines14091912

**Published:** 2026-08-26

**Authors:** Flavio Feri, Yuliya Hutsava, Rita Monico, Francesco Morena, Vincenzo Maria Perriello

**Affiliations:** 1 Department of Rehabilitation Medicine, Santa Maria della Misericordia Hospital, 06132 Perugia, Italy; 2 Department of Life, Health and Environmental Sciences, Link Campus University, 00165 Rome, Italy; 3 Hematology and Bone Marrow Transplantation Unit, Santa Maria della Misericordia Hospital, 06132 Perugia, Italy

**Keywords:** CAR-T cell therapy, autologous stem cell transplantation, physical recovery, rehabilitation, functional mobility, 6-minute walk test (6MWT)

## Abstract

**Introduction**: Chimeric Antigen Receptor T (CAR-T) cell therapy and autologous stem cell transplantation (ASCT) are both associated with significant physical and psychological challenges during post-transfusion recovery. To date, most assessments of physical recovery rely on subjective measures, which are prone to individual perception and bias. Direct comparative data on objective physical function between these therapies remain lacking. **Objective**: The study aims to compare physical recovery trajectories following CAR-T cell therapy and ASCT using objective and standardized rehabilitation assessments to identify promising endpoints for future trials. **Methods**: a total of 331 intraindividual rehabilitation assessments conducted at hospital admission and discharge were retrospectively screened for the Berg Balance Scale (BBS), muscle strength (MS), Timed Up and Go (TUG), 5-Times Sit-to-Stand (5×STS), and 6-minute Walk Test (6MWT). Subjective assessments including self-reported health status and fatigue measured by a Visual Analog Scale were also recorded. **Results**: Twelve patients met the inclusion criteria and completed all rehabilitation assessments (five CAR-T, seven ASCT). In the ASCT group, 6MWT performance declined significantly from admission to discharge (median change −84 m; range −415 to −31 m; *p* = 0.016), while CAR-T recipients showed a non-significant numerical improvement (median change +79 m; range −40 to +148 m; *p* = 0.19). Between-group comparison of 6MWT change favored CAR-T therapy (*p* = 0.005). For BBS, TUG, 5×STS and MS, no statistically significant within-group changes were observed, although trends suggested better preservation of balance and mobility in the CAR-T group. **Conclusions**: This pilot study provides preliminary comparative evidence suggesting that patients receiving CAR-T therapy may better preserve functional endurance than those undergoing ASCT, measured by the 6MWT. These findings are consistent with the existing literature highlighting the sensitivity and clinical relevance of the 6MWT in detecting functional changes, supporting its inclusion in standard rehabilitation assessments.

## 1. Introduction

Chimeric Antigen Receptor T (CAR-T) cell therapy and autologous stem cell transplantation (ASCT) have transformed the therapeutic landscape for relapsed/refractory (r/r) B-cell malignancies, including B-cell Non-Hodgkin Lymphoma (B-NHL) and Multiple Myeloma (MM) [1,2]. However, both therapies impose considerable physical and psychological burdens during the recovery period, including profound fatigue, muscle weakness, impaired mobility, and balance deficits [3,4], hindering patients’ return to daily activities and compromise overall quality of life even in disease remission [5,6].

The recovery process following both CAR-T and ASCT is complicated by multiple treatment-related factors: myeloablative/lymphodepleting chemotherapy, cytokine release syndrome (CRS) and immune effector cell-associated neurotoxicity syndrome (ICANS) in CAR-T recipients [7,8], prolonged cytopenias requiring extended hospitalization, infection risk necessitating isolation and restricted mobility, and systemic inflammation [9,10]. Cumulatively, these factors contribute to rapid physical deconditioning, sarcopenia, and reduced functional independence—effects that may be especially pronounced in older or frail patients [11,12]. Acute functional decline begins during ASCT hospitalization [13], with possible prolonged reductions in exercise capacity [14]. Whether CAR-T recipients experience similar trajectories of decline remains poorly characterized.

Traditionally, physical assessments have been measured using subjective performance status scales such as the Eastern Cooperative Oncology Group (ECOG) score or Karnofsky Performance Status (KPS), quality-of-life questionnaires, and general geriatric assessments [15,16]. While these tools provide valuable information about overall functional status, they are susceptible to inter-rater variability, ceiling effects, and patient reporting bias. Moreover, they may not sensitively capture changes in specific functional domains such as strength, balance, or endurance [17,18]. Standardized, objective rehabilitation tools offer a complementary and potentially more sensitive approach to characterizing functional recovery. The 6-Minute Walk Test (6MWT), Timed Up and Go (TUG), 5-Times Sit-to-Stand test (5×STS), Berg Balance Scale (BBS), and manual muscle strength testing are well-validated instruments extensively used in rehabilitation medicine, cardiopulmonary disease and geriatric populations [19,20]. These measures provide quantitative, reproducible assessments of endurance, mobility, lower limb strength, dynamic balance, and fall risk.

Recent evidence suggests that baseline 6MWT performance may hold prognostic significance in CAR-T recipients [21]. Furthermore, Hamada et al. (2024) found that severity of CRS and occurrence of ICANS were associated with greater declines in 6MWD at 30 days post-CAR-T, indicating that treatment-related toxicities directly impact short-term functional capacity [22]. However, these studies did not include direct comparisons with other intensive therapies such as ASCT.

Despite the increasing use of both CAR-T and ASCT in clinical practice, no robust head-to-head studies comparing objective physical recovery trajectories between these two cellular therapy modalities currently exist. The existing literature on CAR-T rehabilitation consists primarily of small, single-center pilot studies and retrospective cohorts [22,23,24], whereas ASCT rehabilitation research has focused mainly on prehabilitation or long-term survivor outcomes [14,25,26]. As CAR-T cell therapy becomes available earlier in treatment algorithms and for older patient populations [27,28], understanding its comparative functional impact vs. ASCT is increasingly relevant for treatment selection, patient counseling and rehabilitation planning.

The present pilot study aims to directly compare objective physical recovery after CAR-T cell therapy versus ASCT using a comprehensive set of standardized rehabilitation assessments. Specifically, we sought to describe changes in functional endurance, balance, mobility and strength from hospital admission to discharge in CAR-T and ASCT recipients, and to identify which rehabilitation tools are most sensitive for detecting clinically meaningful changes, thereby informing the design of future prospective studies and standardized assessment protocols.

## 2. Methods

This retrospective observational cohort study was conducted in a single tertiary academic medical center with specialized programs in hematologic malignancies and cellular therapies. 

### 2.1. Patient Population

Medical records of 331 adults (≥18 years) with B-NHL or MM who received CAR-T therapy (n = 48) or ASCT (n = 283) at the University Hospital of Perugia between September 2020 and December 2024 were reviewed. Patients were included if they completed comprehensive rehabilitation assessments at admission and discharge for all measures (BBS, TUG, 5×STS, 6MWT, muscle strength). The assessment battery was conducted by trained physiotherapists using validated instruments and standardized administration protocols. Patients were excluded if one or more assessments were missing, unavailable in the medical records, or could not be completed because of severe comorbidities, altered consciousness, orthopedic contraindications, clinical deterioration, or early death. As a result, 12 patients (5 CAR-T and 7 ASCT) met all eligibility criteria and were included in the final analysis.

All CAR-T patients received standard lymphodepleting chemotherapy (fludarabine and cyclophosphamide) followed by transfusion of commercial CAR-T products (axicabtagene ciloleucel, Brexucabtagene autoleucel or tisagenlecleucel). Patients remained hospitalized from the start of lymphodepletion through approximately 3–4 weeks post-transfusion for monitoring and management of potential toxicities including CRS and ICANS, according to institutional protocols [29]. ASCT patients underwent peripheral blood stem cell mobilization, high-dose chemotherapy conditioning (melphalan 200 mg/m^2^ IV for MM or FEAM regimen–Fotemustine 150 mg/m^2^ on day −8, Etoposide 200 mg/m^2^ IV twice daily on days −7 to −4, Cytarabine 200 mg/m^2^ IV twice daily on days −7 to −4 and Melphalan 140 mg/m^2^ IV on day −1 for B-NHL), followed by autologous stem cell transplantation. Patients remained hospitalized through neutrophil engraftment and resolution of acute toxicities.

### 2.2. Objective Assessment Tools

1. *Muscle Strength Testing.* Manual muscle testing was performed for major muscle groups considered critical for functional mobility and independence, including hip flexors, knee extensors, ankle dorsiflexors, shoulder abductors, and elbow flexors. Strength was graded using the standard 0–5 Medical Research Council (MRC) scale [30].
2. *TUG.* The TUG assesses functional mobility, balance (both static and dynamic), and fall risk [31]. Patients were instructed to stand from a seated position in a standard armchair, walk 3 m at a comfortable pace, turn around, return to the chair, and sit down. Time to completion was recorded in seconds. A cutoff of >13.5 s has been associated with increased fall risk in older adults [32].
3. *5×STS.* This test measures lower limb muscle strength, power, and functional capacity [33]. Patients were asked to stand up and sit down from a standard chair five times as quickly as possible without using their arms. The total time to complete five repetitions was recorded and compared to age- and sex-specific normative values [34].
4. *BBS.* The BBS is a 14-item objective measure assessing static and dynamic balance abilities across various functional tasks [35]. Each item is scored from 0 (unable to perform) to 4 (performs independently), yielding a total score of 0–56. Scores below 45 indicate a greater risk of falls [36].
5. *6MWT.* The 6MWT measures functional exercise capacity and endurance [37]. Patients walked at their fastest comfortable pace along a flat, marked 30 m corridor for 6 min, with standardized encouragement provided at 1 min intervals. The total distance covered (in meters) was recorded. Baseline 6MWD was calculated as a percentage of predicted distance using validated reference equations accounting for age, sex, height, and weight [38].

### 2.3. Subjective Assessment Tools

1. *Self-Reported Health Status*. Patients rated their overall perceived health status on a visual analog scale ranging from 0 (worst imaginable health) to 100 (best imaginable health).
2. *Visual Analog Scale (VAS) for Fatigue*. Fatigue was assessed using a 0–10 VAS, with 0 representing no perceived exertion and 10 representing maximal exertion or exhaustion.

### 2.4. Data Collection and Management

All assessment data were recorded on standardized forms and subsequently entered into a secure electronic database. Data were anonymized prior to analysis. Demographic information (age, sex, body mass index), disease characteristics (diagnosis, disease stage, prior treatment lines), treatment details (CAR-T product or ASCT conditioning regimen), and clinical outcomes (length of stay, occurrence of CRS/ICANS for CAR-T, major complications) were extracted from electronic medical records.

### 2.5. Statistical Analysis

Data were anonymized and analyzed using GraphPad Prism version 10 (GraphPad Software, Boston, MA, USA). Given the very small sample size and the exploratory nature of the study, we did not assume normal distribution of the functional parameters. Data distribution was evaluated descriptively and non-parametric tests were selected as more appropriate in this context. Within-group changes were assessed using the Wilcoxon signed-rank test. Between-group differences (CAR-T vs. ASCT) at admission, at discharge, and in change scores (Δ admission–discharge) were evaluated using the Mann–Whitney U test for continuous variables and Fisher’s exact test for categorical variables where appropriate. Given the small sample size and exploratory design, *p*-values < 0.05 were considered statistically significant without adjustment for multiple comparisons.

## 3. Results

### 3.1. Patient Characteristics

A total of 12 patients met inclusion criteria and comprised the analytical cohort: five patients who received CAR-T cell therapy (two Tisagenlecleucel, two Brexucabtagene autoleucel and one Axicabtagene ciloleucel) and seven patients who underwent ASCT. All CAR-T recipients had B-NHL, whereas the ASCT group included both B-NHL (57%) and MM (43%) patients. Baseline demographic and clinical characteristics are presented descriptively owing to the limited sample size (Table 1).

### 3.2. Functional Assessment Outcomes

**Figure 1:**
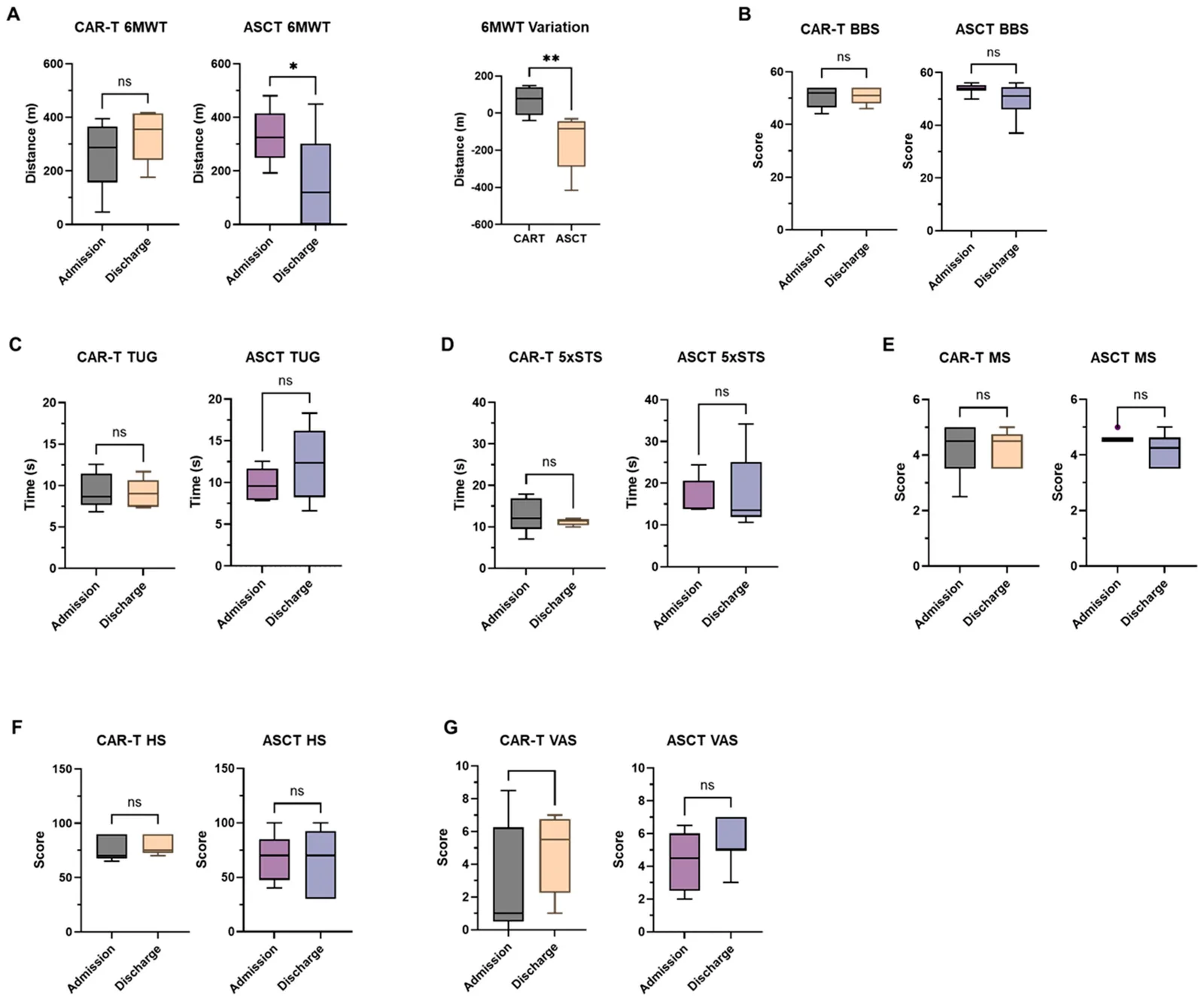
Objective rehabilitation outcomes in patients undergoing CAR-T cell therapy or ASCT. (**A**) Six-minute walk distance (6MWD): CAR-T patients showed preserved or slightly improved endurance, whereas ASCT patients experienced a significant decline, with a larger reduction in 6MWD. (**B**) Berg Balance Scale (BBS): balance remained globally preserved in both groups, with all scores above the fall-risk threshold. (**C**) Timed Up and Go (TUG): mobility was stable after CAR-T but tended to worsen after ASCT, with a significant between-group difference in change. (**D**) Five-Times Sit-to-Stand (5×STS): only small, non-significant variations were observed. (**E**) Manual muscle strength: no clinically relevant strength loss was detected. (**F**,**G**) Patient-reported health status and fatigue: CAR-T patients showed a trend toward better perceived health and less fatigue worsening than ASCT recipients, without statistically significant differences. * for *p* < 0.05; ** for *p* < 0.005.

Comprehensive rehabilitation assessment results at admission and discharge are summarized in Table 2 and Figure 1.

Patients undergoing ASCT demonstrated a significant decline in 6MWT performance during hospitalization. Median 6MWD decreased from 325 m (range 192–480 m) at admission to 120 m (range 0–449 m) at discharge (*p* = 0.016), with a median within-patient change of −84 m (range −415 to −31 m). One ASCT recipient was unable to complete the 6MWT at discharge (6MWD = 0 m), reflecting severe fatigue and deconditioning. In contrast, patients receiving CAR-T cell therapy showed a non-significant numerical improvement in 6MWD, from a median of 287 m (range 46–395 m) at admission to 355 m (range 176–416 m) at discharge (*p* = 0.19; median change +79 m, range −40 to +148 m). While baseline 6MWD values were similar between groups (*p* = 0.53), the median change in 6MWD differed significantly between CAR-T and ASCT groups from admission to discharge (+79 m vs. −84 m, respectively; *p* = 0.005), indicating that ASCT recipients experienced substantially greater functional decline during hospitalization (Figure 1A).

Balance performance, assessed by the BBS, was high in both groups at admission and remained largely preserved at discharge. Median BBS scores were 54 (range 44–54) vs. 55 (50–56) at admission for CAR-T and ASCT, and 52 (46–54) vs. 49 (37–56) at discharge, respectively, with no statistically significant within-group changes. All patients maintained BBS scores above the fall-risk threshold of 45 at both time points (Figure 1B).

TUG performance showed a trend toward slower times at discharge in the ASCT group, whereas CAR-T patients remained relatively stable. Median TUG times in the CAR-T cohort were 8.7 s (6.8–12.5 s) at admission and 9.0 s (7.3–11.7 s) at discharge (*p* = 0.69), compared with 10.0 s (7.8–12.5 s) and 12.5 s (6.6–18.3 s) in the ASCT group (*p* = 0.09). The between-group difference in TUG change reached statistical significance (*p* = 0.03), indicating greater mobility deterioration among ASCT recipients (Figure 1C).

5×STS times showed small, non-significant changes in both groups. CAR-T recipients improved slightly from a median of 12.5 s (7.1–17.9 s) to 11.7 s (9.0–12.1 s; *p* = 0.25), whereas ASCT patients showed no meaningful change (15.0 s [13.8–24.4 s] vs. 15.0 s [10.7–34.2 s]; *p* = 0.88). Between-group differences in 5×STS change were not statistically significant (*p* = 0.11). Manual muscle (MM) testing did not reveal clinically relevant changes in global strength in either cohort, with most muscle groups remaining in the 4–5/5 range throughout hospitalization (Figure 1D,E).

**Table 2:** Functional parameters at admission and discharge in patients with hematologic malignancies undergoing CAR-T cell therapy or ASCT.

Measure	CAR-T Group (n = 5)	ASCT Group (n = 7)	*p*-Value
6-MWD (meters)			
Admission, median (range)	287 (46–395)	325 (192–480)	0.53
Discharge, median (range)	355 (176–416)	120 (0–449)	0.10
Change, median (range)	+79 (−40 to +148)	−84 (−415 to −31)	0.005
Within-group *p*-value	0.19	0.016	
BBS (0–56)			
Admission, median (range)	54 (44–54)	55 (50–56)	0.40
Discharge, median (range)	52 (46–54)	49 (37–56)	0.09
Change, median (range)	0 (−1 to +2)	−1 (−18 to 0)	0.04
Within-group *p*-value	0.79	0.06	
TUG (seconds)			
Admission, median (range)	8.7 (6.8–12.5)	10 (7.8–12.5)	0.45
Discharge, median (range)	9 (7.3–11.7)	12.5 (6.6–18.3)	0.08
Change, median (range)	−0.4 (−1.3 to +1)	+1.5 (−1.4 to +7.5)	0.03
Within-group *p*-value	0.69	0.09	
5×STS (seconds)			
Admission, median (range)	12.5 (7.1–17.9)	15 (13.8–24.4)	0.3
Discharge, median (range)	11.7 (9–12.1)	15 (10.7–34.2)	0.12
Change, median (range)	−1 (−7 to +2.9)	0 (−3.1 to +9.8)	0.11
Within-group *p*-value	0.25	0.88	

### 3.3. Subjective Assessments

Both groups reported moderate perceived health status at admission (median scores of 60–65/100), with slight improvements at discharge in the CAR-T group (median 70/100) but continued decline in the ASCT group (median 50/100). These trends were not statistically significant given the small sample size. Fatigue scores increased in both groups from admission to discharge, reflecting the acute physical demands of treatment. Median fatigue VAS increased from 4/10 to 6/10 in the CAR-T group and from 5/10 to 7/10 in the ASCT group (*p* > 0.05 for both) (Figure 1F,G).

## 4. Discussion

Incorporating systematic functional assessments into routine care may be particularly relevant as CAR-T therapy moves earlier in treatment algorithms and is offered to older and more frail patients who may be especially vulnerable to functional decline [39]. For patients eligible for both CAR-T cell therapy and ASCT, considering the potential for better short-term preservation of functional endurance with CAR-T may contribute to shared decision making, alongside classical oncologic outcomes and toxicity profiles [40].

This pilot study provides preliminary comparative data on short-term functional recovery during hospitalization in patients receiving CAR-T cell therapy or ASCT using a comprehensive battery of standardized rehabilitation assessments. While ASCT patients experienced a median decline of −84 m in the 6MWT from admission to discharge, consistent with acute functional deconditioning during high-dose chemotherapy and the aplastic phase, CAR-T cell recipients maintained stable functional capacity with no significant change in their 6MWD performance. In contrast, other objective measures such as balance (BBS), mobility (TUG), and muscle strength did not show significant changes in either group, suggesting that these tools may be less sensitive than the 6MWT for capturing short-term functional variations in this specific clinical context. Although the limited sample size and exploratory nature of this pilot study preclude definitive conclusions, our findings suggest that the 6MWT may be the most sensitive assessment for detecting short-term functional changes during hospitalization, supporting its further evaluation in future prospective studies.

Several factors likely contribute to the observed differences only in functional endurance, suggesting that the physiological stress and immobilization associated with ASCT may have a more profound acute impact than CAR-T cell therapy. ASCT typically requires high-dose myeloablative chemotherapy, such as high-dose melphalan [41], resulting in deeper and more prolonged cytopenias, substantial mucosal toxicity [42], infectious complications and delayed hematologic recovery. These treatment-related effects can contribute to more sustained tissue damage, prolonged fatigue and muscle catabolism, thereby exacerbating functional decline during hospitalization [43]. Conversely, the lymphodepleting chemotherapy used in CAR-T therapy (fludarabine and cyclophosphamide) is non-myeloablative and substantially less intensive, with shorter cytopenic periods that may permit earlier mobilization [44]. In addition, while CAR-T therapy can trigger CRS and ICANS, these toxicities are generally time-limited and effectively managed with tocilizumab and corticosteroids, potentially mitigating their impact on physical function [24].

Our results align with and extend existing literature on physical function in cellular therapy recipients, highlighting that the 6MWT is a particularly sensitive and clinically relevant tool for detecting functional changes in cellular therapy recipients. Hamada et al. recently demonstrated that CRS severity and the occurrence of ICANS in CAR-T cell recipients are associated with short-term declines in 6MWD at 30 days, highlighting the sensitivity of 6MWT to capture treatment-related functional impairment [22]. Similarly, McCourt et al. and McGoldrick et al. reported that structured prehabilitation and rehabilitation programs are feasible and may help preserve physical fitness in both CAR-T and hematopoietic stem cell transplant recipients, although most prior studies have focused on single cohorts without direct comparison between treatment modalities [13,24].

In this context, our head-to-head evaluation using identical rehabilitation protocols for CAR-T and ASCT within the same institution provides complementary information and helps identify which objective measures may be most informative for future trials. Specifically, our data support the notion that the 6MWT is sensitive to short-term differences in endurance between these therapies, whereas balance and strength measures remained relatively preserved in both groups. Future research involving larger, prospective multicenter cohort studies with longer follow-up periods is required to validate the integration of the 6MWT integration into standard pre- and post-treatment assessment protocols to inform risk stratification and personalize rehabilitation needs throughout the treatment journey. For patients eligible for both therapies, such as those with relapsed diffuse large B-cell lymphoma, the potential for better functional preservation with CAR-T cells may become an important aspect of shared decision making, especially for older or frailer individuals, once confirmed by adequately powered studies.

Although our study provides head-to-head objective functional data within a single institution using identical protocols, thereby minimizing procedural heterogeneity, several important limitations must be acknowledged. The small sample size limits statistical power and the ability to control for confounders such as age or disease burden. Such limited number of evaluable patients was related to the requirement for complete rehabilitation assessments, since not all patients undergoing CAR-T therapy or ASCT completed the entire battery of tests because of logistical and/or clinical constraints during hospitalization. Consequently, only patients with complete and comparable rehabilitation data could be included in the final analysis. Furthermore, the retrospective, single-center design introduces inherent risks of bias and does not allow control over rehabilitation timing, intensity or adherence. The underlying hematologic malignancies also differed between groups and we did not adjust for disease related factors, comorbidity burden or baseline frailty, all of which may influence functional trajectories. Finally, limiting the study only to hospital assessments did not capture long-term recovery beyond discharge, preventing any inference about persistent or late functional differences.

Overall, this pilot study should be viewed as a preliminary evidence towards incorporating the 6MWT into larger, methodologically robust comparison between CAR-T and ASCT recipients to confirm whether the observed differences persist over time, translate into meaningful quality-of-life benefits, and can ultimately inform individualized supportive care and rehabilitation pathways in cell therapy practice.

## Figures and Tables

**Table 1:** Baseline patient characteristics.

Characteristic	CAR-T Group (n = 5)	ASCT Group (n = 7)
Age, years		
Median (range)	58 (48–64)	53 (42–64)
Sex, n (%)		
Male	2 (40%)	3 (60%)
Female	3 (60%)	4 (40%)
ECOG, n (%)		
0–1	4	7
>2	1	0
Body Mass Index, kg/m^2^		
Median (range)	26.98 (19.98–30.96)	28.39 (19.3–40.27)
Length of Stay, days		
Median (range)	26.8 (24–41)	23.6 (19–31)

## Data Availability

The data that support the findings of this study are available from the corresponding author upon reasonable request, subject to institutional privacy and ethics regulations. The data are not publicly available due to ethical restrictions imposed by the Ethics Committee and privacy regulations regarding patient data.
